# The effects of esketamine and treatment expectation in acute major depressive disorder (Expect): study protocol for a pharmacological fMRI study using a balanced placebo design

**DOI:** 10.1186/s13063-023-07556-x

**Published:** 2023-08-11

**Authors:** Irina Falkenberg, Florian Bitsch, Wei Liu, Alexandros Matsingos, Laila Noor, Christoph Vogelbacher, Cüneyt Yildiz, Tilo Kircher

**Affiliations:** 1 Department of Psychiatry and Psychotherapy, University of Marburg, Rudolf-Bultmann-Str. 8, 35039 Marburg, Germany; 2 Center for Mind, Brain and Behavior - CMBB, Hans-Meerwein-Straße 6, 35032 Marburg, Germany; 3 Department of Clinical Psychology, University of Marburg, Schulstr. 12, 35037 Marburg, Germany

**Keywords:** Expectation, Esketamine, Major depression, Placebo, fMRI

## Abstract

**Background:**

Major depressive disorder (MDD) is a highly prevalent (8–15%), severely disabling disorder and is associated with enormous socioeconomic impact. Antidepressant medication for the treatment of MDD has proven effective in RCTs; however, placebo response is also substantial. Given the potential benefits of modulating the placebo response in patient care and pharmacological research, understanding the mechanisms underlying placebo response is of high clinical relevance. The placebo response is mediated by treatment expectation, i.e. an individual’s belief about whether and how much they will improve as a consequence of their treatment. The mechanisms and moderators of treatment expectation effects in MDD are poorly understood. Initial brain imaging studies on placebo responses in MDD point towards the relevance of the lateral prefrontal cortex and the rostral anterior cingulate cortex (rACC). In this project, we will investigate the neural mechanisms underlying the antidepressant effects of treatment expectation associated with the fast-acting antidepressant esketamine in patients with MDD. Esketamine is an NMDA receptor antagonist inducing antidepressant effects within hours.

**Methods:**

We will employ a fully balanced placebo design with the factors “treatment” (i.v. esketamine / placebo) and verbally induced “expectation” (high / low) combined with fMRI (resting state, emotion and reward processing paradigms) to investigate the psychological and neural mechanisms underlying the antidepressant effects of expectation, and how these interact with the pharmacological effects of esketamine.

**Discussion:**

The insights gained by this project promise fundamental implications for clinical treatment and future drug trials. Unraveling the mechanisms underlying expectation effects on antidepressant treatment may inform (1) strategies to modulate these effects and thus improve assay sensitivity in RCTs and (2) novel treatment regiments aiming to maximize the synergistic effects of expectation and pharmacological treatment in the clinical care of patients with MDD.

**Trial registration:**

This trial has been prospectively registered with the EU Clinical Trials Register: EudraCT-No.: 2020–000784-23 (November 17, 2020).

## Introduction

### Background and rationale {6a}

Major depressive disorder (MDD) has a lifetime prevalence of around 8–15%, affecting about 300 million people worldwide. MDD is the leading global cause of disability in terms of total years lost due to disability and is associated with excess mortality [1]. About one third of patients with MDD fail to achieve remission despite treatment with multiple antidepressants and are considered to have treatment-resistant depression [2]. Treatment algorithms suggest that an antidepressant medication be started initially at lower doses and that adjustment of the medication maybe considered if at least a moderate improvement is not observed after 4–6 weeks. This means that even in patients who do respond to antidepressants the time to onset of effect can be several weeks. During this time, patients may remain symptomatic and at risk of suicidal behaviour. An unresolved issue in antidepressant trials are high placebo responses which has been suspected to contribute to so-called failed antidepressant trials [3]. Patients’ expectation about treatment benefits is thought to play a major role in the placebo response [4]. The neural circuits involved in the effects of treatment expectation on pain and affective processing include limbic and reward-related brain areas (e.g. amygdala, rostral anterior cingulate cortex striatum) as well as the prefrontal cortex [5, 6]. Knowledge of the differential neurobiological mechanisms underlying the response to pharmacological treatments vs the effects of positive expectation in MDD is almost completely lacking. Better knowledge of such mechanisms would significantly improve our understanding of antidepressant effects and ultimately help us to provide more effective relief of depressive symptoms for patients.

#### Functional neuroimaging of emotion and reward processing in MDD

Most patients with MDD present with low mood and anhedonia (i.e. loss of pleasure and blunted responsiveness to reward). The severity of these cardinal symptoms of MDD is related to emotion dysregulation (low mood) and deficits in reward value processing (anhedonia) [7]. Structural and functional magnetic resonance imaging (MRI) studies in MDD (irrespective of treatment) vs healthy subjects have identified distinctive morphological and functional brain changes in MDD mainly in cortico-limbic networks [8, 9]. Brain regions involved in these networks, in particular DLPFC, insula, rACC, and amygdala, are crucial for the processing of information with emotional (e.g. emotional faces) or motivational (e.g. monetary rewards) significance. Functional imaging data acquired while patients with MDD process emotional stimuli have widely demonstrated altered physiological responses in the amygdala as well as in anatomically related areas [10], with negative emotional stimuli (e.g. fearful faces) eliciting enhanced amygdala responses in patients [8], while amygdala hypo-activity has been found in relation to positive facial expressions [8, 9]. The brain reward system (BRS) mediates reward behaviours, pleasure, and motivation and has been associated with anhedonia [11]. A number of fMRI studies have been conducted in order to elucidate brain reward processes involved in MDD [11, 12]. For example, using a monetary incentive delay task in adults with or without MDD, one study found relatively reduced putamen activation during reward anticipation, reduced activation in nucleus accumbens (Nacc) and caudate during receipt of reward [13] and increased ACC activation during anticipation of monetary gains in the MDD group [14]. It is yet unclear how alterations in the emotional and reward systems are related to treatment expectation in MDD.

#### Neural-functional targets of antidepressant treatment and placebo in MDD

Functional MRI studies have helped to identify brain regions responding to antidepressant treatments in healthy people and patients with MDD. Generally, these regions are part of emotion- and reward-processing networks, i.e. DLPFC, insula, ACC and amygdala [15]. More specifically, antidepressant treatment (mainly SSRI and SNRI) has been found to normalize abnormally elevated responses to mainly negative emotional stimuli particularly in the amygdala but also other limbic areas (e.g. ACC), to enhance PFC activity and to enhance the coupling between subcortical (amygdala, thalamus, striatum) and cortical (ACC, PFC) regions in patients with MDD (see [15] for a review). Yet, it is unclear how drug-specific effects separate from other non-specific elements of the treatment response, such as the placebo effect [16]. This is important, as placebo response rates in antidepressant clinical trials average 35–40% compared with response rates to antidepressants of around 50% and there is some evidence that the placebo response rate increases with increasing publication year [3], although this is subject to debate [3].

While pioneering studies have started to reveal the neural basis of placebo effects in emotional processing in healthy volunteers (see [17] for a review), the mechanisms underlying the antidepressant effects of expectation induced by placebo treatments in MDD are largely unexplored. Studies of the neural mechanisms underlying placebo effects in antidepressant treatment have largely been limited to demonstrating differences in brain activity between responders and non-responders to placebo. The few available studies on placebo responses in MDD point towards a contribution of the PFC and rACC [6, 18, 19]; however, their naturalistic design cannot support identification of the mechanisms underlying placebo effects, such as patient outcome expectation [20]. To date, only one few randomized controlled studies designed to identify the neural mechanisms of expectation augmentation in antidepressant treatment have been published. An SSRI randomized control trial has been published. The results of the first study [21] suggested that manipulating outcome expectation through increasing the probability of receiving active medication (SSRI vs placebo) was associated with decreased amygdala activation in response to sad emotional faces, which in turn was associated with more rapid reduction in depressive symptoms during the course of later antidepressant treatment. However, the sample size of this study was small (total *N* = 23 patients, only 4 patients randomized to placebo condition). A larger study (*N* = 66) using a similar design aimed to identify baseline neuroimaging and cognitive predictors of response to expectancy effects in elderly outpatients with MDD [22]. Patients benefiting from the manipulation in terms of greater antidepressant treatment response showed greater processing speed, executive function and frontostriatal white matter tract integrity. Antidepressant medication was, however, heterogeneous in this trial and the designs of both studies did not allow to differentiate between expectation related antidepressant effects and unspecific effects (e.g. natural history, additional psychological therapies) due to the delay between expectation induction and actual antidepressant or placebo treatment. In order to develop novel treatment strategies for patients which provide effective symptom relief it is crucial to understand expectation mechanisms early in the treatment process, ideally after a single-dose of a fast-acting antidepressant, which may help to identify responders to antidepressant or placebo treatment early, with the benefit of avoiding unnecessary dose increases and side effects. Based on these considerations, we will use esketamine as a fast-acting compound in a large sample of MDD patients (*N* = 176) which will allow us to perform the expectation manipulation and the measurement of its effects on the same day.

#### Fast-acting antidepressant esketamine: clinical utility and pharmacological MRI

Besides serotonergic, noradrenergic (emotion processing) and dopaminergic (reward processing) systems, abnormalities in glutamatergic neurotransmission have been implicated in the pathophysiology of MDD. Importantly, the N-methyl-D-aspartate (NMDA) receptor antagonist ketamine—which has been in use as an anaesthetic for decades—has antidepressant effects in subanaesthetic doses within hours. This rapid effect distinguishes it from conventional compounds which induce a clinically relevant effect only after 1–3 weeks. A recent meta-analysis of randomized, placebo/pseudo-placebo-controlled trials of single-dose, i.v. ketamine or non-ketamine NMDAR antagonists for treatment-resistant patients with MDD and bipolar depression has shown that single ketamine infusion was significantly superior to placebo/ pseudo-placebo regarding antidepressant efficacy [23]. The significantly greater reduction in depressive symptoms started within 40–60 min, peaked on day 1 and lasted until days 5–8, with maintenance of superior remission and response status until days 3–5 and 7, respectively. Effect sizes ranged from small to large (− 0.38 to − 1.00) for symptom reduction (large for response [NNT = 2–5, peaking at 230–240 min] and remission [NNT = 3–7, peaking at 1 day]).

Recently, a novel route of administration has also been introduced. Esketamine, the S-enantiomer of ketamine with a higher affinity for the NMDA receptor than the R-enantiomer, can be applied as a nasal spray. Following intranasal application of esketamine, robust and durable antidepressant efficacy has been demonstrated in treatment-resistant MDD [24]. The neural signaling changes induced by administration of sub-anaesthetic esketamine can be measured using pharmacological magnetic resonance imaging (phMRI). The existing studies in patients with MDD have used ketamine i.v.; no phMRI study using esketamine in patients with MDD has been published so far. PhMRI studies aiming to identify the neural correlates of ketamine treatment by use of (resting state) fMRI, diffusion tensor imaging (DTI), magnetic resonance spectroscopy (MRS) and magnetoencephalography (MEG) show that ketamine produces robust and consistent effects throughout the entire brain (see [25] for a review). A few phMRI studies using various methods (MEG, rsfMRI, fMRI, DTI, MEG, MRS) have focused on ketamine effects on brain networks relevant for emotion processing and suggest that ketamine alters activity in and increases functional connectivity between regions such as the right lateral PFC, sgACC, bilateral amygdala, NAcc, hippocampus and thalamus [26–29], with ketamine-induced changes correlating with the antidepressant effect. Only a limited number of studies have specifically focused on investigating ketamine’s impact on brain regions responsible for reward processing. One study has used fMRI to investigate the effects of ketamine i.v. (without placebo) on the functioning of neural networks specifically related to reward processing in 10 patients with treatment-resistant MDD [30]. This study found that mood improvement was accompanied by an increased recruitment of the orbitofrontal cortex, ventral striatum, medial substantial nigra and ventral tegmental area, structures that are part of the reward system. Using an ROI-based approach, a second study found a significant drug effect particularly in the nucleus accumbens and putamen in 37 unmedicated remitted patients with MDD during a Monetary Incentive Delay Task (MID; [31, 32]). Additional evidence for ketamine’s modulatory effects on brain regions involved in reward processing was provided by a study showing that ketamine reduced sgACC hyper-activation to positive incentives in 28 patients with MDD, indicating a normalizing effect of ketamine on aberrant sgACC functioning [33].

The existing fMRI studies on ketamine mechanisms in MDD generally have (i) small sample sizes, (ii) usually no placebo condition, (iii) only included treatment-resistant patients, (iv) not used esketamine in MDD, (v) only used i.v. administration and, most importantly, (v) did not investigate expectation effects which are very likely to contribute to the success of a novel treatment approach such as esketamine. Taken together, the rapid antidepressant effect and its robust effects on brain regions critically relevant for the pathophysiology of MDD make application of esketamine vs placebo an ideal model to disentangle treatment and treatment expectation effects at the neural and the behavioural level in MDD.

### Objectives {7}

The aim of this study is to provide evidence for the effects of positive expectation (high/low) on the neural and behavioural correlates of response to antidepressant treatment in patients with MDD. We will manipulate outcome expectation by disclosing different probability rates (high probability = 90%, low probability = 10%) of receiving a fast-acting antidepressant (i.v. esketamine) vs placebo in this randomized controlled trial. Using functional and structural MRI techniques, we will assess the effects of expectation manipulation, pharmacological treatment and the interaction thereof on the neural processes related to core MDD features, namely emotional dysregulation (Study 1: Emotion processing) and anhedonia (Study 2: Reward processing). The study will be performed as part of the Collaborative Research Center (CRC) SFB / TRR 289 “Treatment expectation”. This CRC aims to investigate the role of patients’ expectations about treatment benefits as important modulators of health outcomes. The ultimate goal of the CRC is to generate the knowledge base for the systematic utilization of patients’ expectations in order to optimize therapeutic strategies and thereby improve health outcomes. In a highly interdisciplinary and translational effort, the CRC will investigate the psychological and neurobiological mechanisms of as well as the interindividual differences in the effects of expectation on health outcomes. The present study will therefore also provide standardized behavioural, (f)MRI data for pooled and meta-analytic approaches within the CRC to identify predictors of interindividual differences in the effects of expectation on health outcomes.

### Trial design {8}

A double-blind, randomized, controlled parallel-group, 4-arm, monocentre trial using a balanced placebo design in a pharmacological fMRI study and exploratory framework.

## Methods: participants, interventions and outcomes

### Study setting {9}

**Fig. 1:**
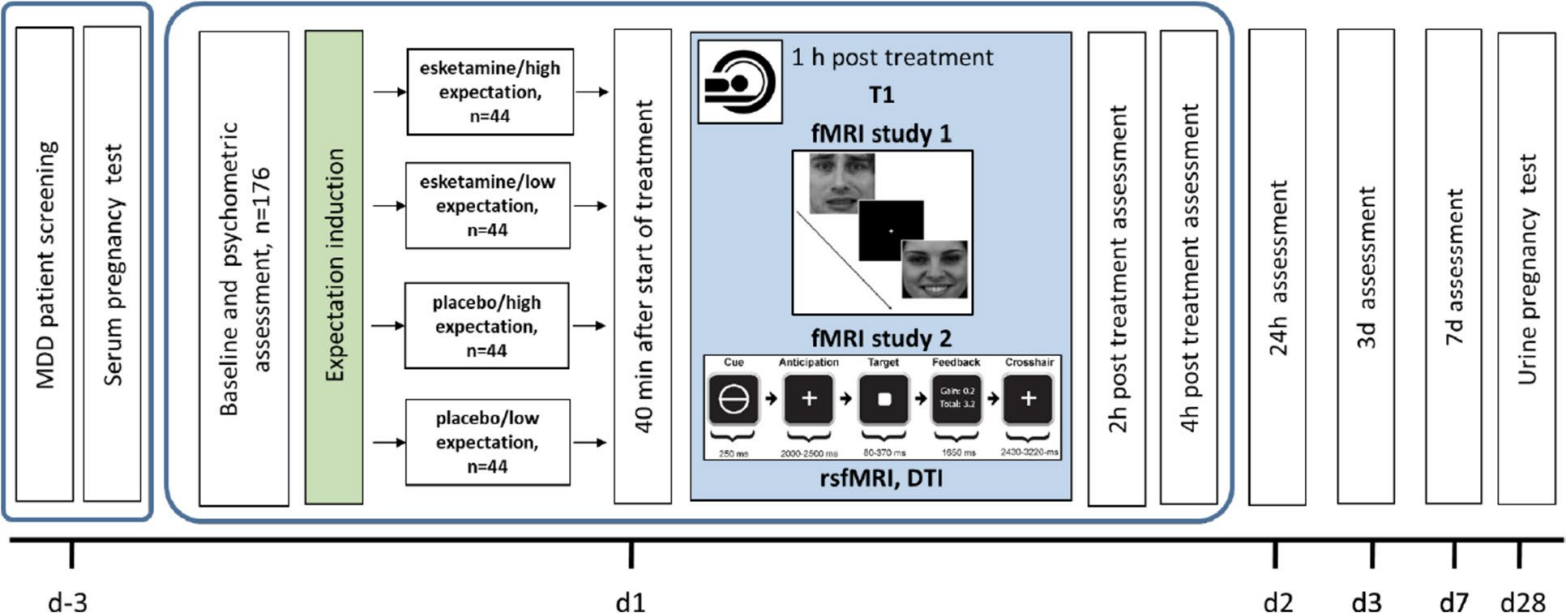
Study design. One hundred seventy-six patients with acute MDD will be recruited (d-3), undergo baseline assessment (d0_1_), female participants of child bearing age will undergo a serum pregnancy test (d-3). At d1, patients will be randomized into 4 groups with the factors treatment (esketamine/placebo) and expectation (high/low). They will take part in two fMRI studies, rsfMRI, DTI and T1 scanning. Clinical outcomes will be assessed after 2 h, 4 h (all d1), 24 h (d2), 3d and 7d post treatment. Urine pregnancy tests will be performed at day 28

A total of 176 patients with MDD according to DSM-5 criteria will be recruited from in- and outpatient settings from the Department of Psychiatry and Department of Psychology, University of Marburg, the Department of Clinical Psychology, University of Marburg and surrounding psychiatric hospitals. The diagnosis will be confirmed by a specialized psychiatrist or psychologist using the Structured Clinical Interview for DSM-5 (SCID-5). All patients will visit the lab 6 times (see Fig. 1 and Table 1, first and last 2 visits possible as telephone interviews in outpatients) and will be financially compensated for participation. All participants will have to be on a stable antidepressant medication for at least 2 weeks.

**Table 1:**
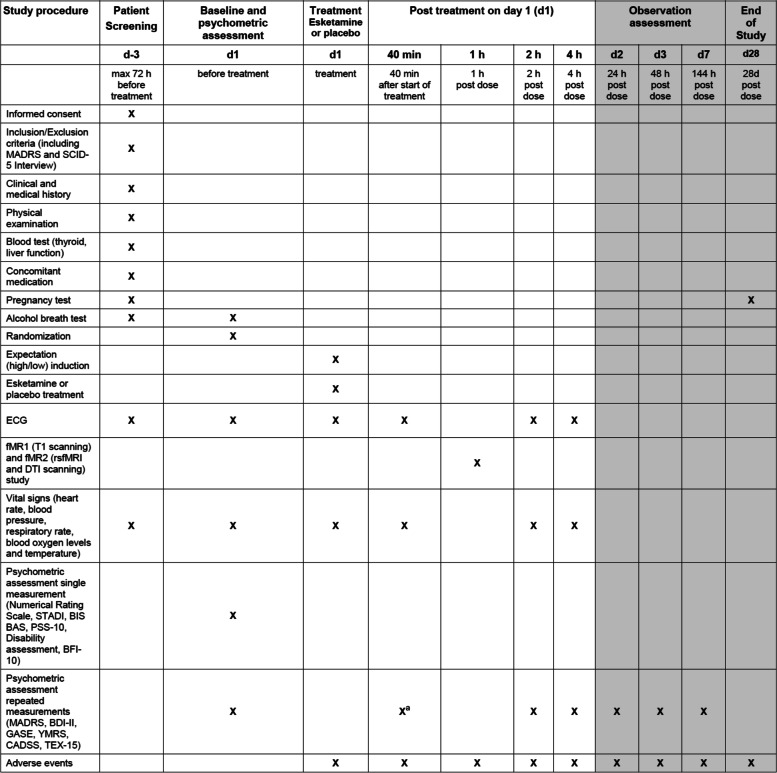
SPIRIT participant timeline fort he EXPECT study

^a^BDI-II and GASE only

### Eligibility criteria {10}

#### Inclusion criteria

1. A current depressive episode lasting at least four weeks
2. An initial score of at least 7 (corresponding to a mild degree of depression) on the Montgomery Åsberg Depression Rating Scale (MADRS)
3. Negative serum pregnancy test in women of childbearing potential
4. Patients with reproductive potential must agree to maintain highly effective methods of contraception by practicing abstinence or by using at least two methods of birth control from the date of consent through the end of the study. If abstinence could not be practiced, a combination of hormonal contraceptive (oral, injectable or implants) and a barrier method (condom, diaphragm with a vaginal spermicidal agent) has to be used.

#### Exclusion criteria

1. Psychotic symptoms (ascertained using SCID-5 Interview)
2. Suicidality (clinical assessment by study physician)
3. Hypertension > 180/100 mmHg (according to resting blood pressure, as assessed by study physician using blood pressure measurement)
4. Hyperthyroidism (clinical history by study physician and current thyroid parameters will have to be within the following ranges: TSH 0.34–5.6 mU/l, fT3 3.2–6.9 pmol/l, fT4 7.5–21 pmol/l)
5. Hepatic dysfunction (clinical history by study physician and current liver parameters will have to be within the following ranges: GOT < 35 U/l; GPT < 35 U/l; GGT < 40 U/l, bilirubin 0.1–1.2 mg/dl)
6. Hypersensitivity to the active substance of esketamine or to any of the excipients listed in Sect. 6.1 of the SmPC (clinical history by study physician)
7. Unstable angina or myocardial infarction in the last 6 months (clinical history by study physician)
8. Myocardial failure (clinical history and physical examination by study physician)
9. Glaucoma or perforating eye injuries (clinical history by study physician)
10.  Patients will be excluded if they have any drug or alcohol dependency/abuse within the previous 3 months or if they are under acute influence of alcohol (clinical assessment by study physician and alcohol breath test)
11.  Any contraindications for MRI, i.e. non-removable medical devices (such as pacemakers, insulin pumps, implantable drug infusion pumps) or metal devices /foreign bodies (such as aneurysm clips, metal splinters in the eye, intrauterine devices) and pregnancy (clinical assessment by study physician and serum pregnancy test)
12.  Medical conditions likely to affect brain anatomy or physiology (clinical assessment by study physician)
13.  Age < 18 or > 65 years
14.  Inability to provide written informed consent
15.  Breastfeeding (clinical history by study physician)
16.  Simultaneous participation in other clinical trials if not permitted by the Principal Investigator
17.  Patients for whom an elevated blood pressure or an increased intracranial pressure represents a serious risk will be excluded (clinical history and blood pressure measurement by study physician)
18.  Patients with manifest ischemic heart diseases will be excluded (clinical history by study physician)
19.  Increased intracranial pressure (clinical history by study physician)
20.  Severe psychological disorders other than depression (Structured Clinical Interview for DSM-5 (SCID-5))
21.  Concomitant therapy with Xanthin-derivates and ergometrin (clinical history by study physician)
22.  Treatment with strong inhibitors or inducers of CYP3A4 (for example, but not exclusively, HIV protease inhibitors, macrolide antibiotics, azole antifungals, carbamazepine, phenobarbital (clinical history by study physician))
23.  Benzodiazepine treatment (clinical history by study physician)

### Who will take informed consent? {26a}

Study physicians will inform the patients and obtain the consent for the trial.

### Additional consent provisions for collection and use of participant data and biological specimens {26b}

Not applicable as this trial does not involve collecting biological specimens for storage.

## Interventions

### Explanation for the choice of comparators {6b}

Antidepressant medication for the treatment of MDD has proven effective in RCTs; however, placebo response is also substantial. Given the potential benefits of modulating the placebo response in patient care and pharmacological research, understanding the mechanisms underlying placebo response is of high clinical relevance. The placebo response is mediated by treatment expectation, i.e. an individual’s belief about whether and how much they will improve as a consequence of their treatment. The mechanisms and moderators of such treatment expectation effects in MDD are poorly understood and will be investigated in this study. To this end, placebo (sodium chloride 0.9%) will be used as a comparator.

### Intervention description {11a}

#### Study 1: Emotion processing

The first fMRI study aims to characterize the effects of positive expectation (high vs low) and esketamine (verum vs placebo) on neural circuits underlying emotion processing in patients with MDD. To this end, the patients will perform an implicit emotion processing task involving positively and negatively valenced stimuli (happy and fearful faces) and indicate the gender of the person displayed by button press (for details, see “fMRI paradigms” below and Fig. 1) after having received the expectation induction followed by the study medication. Measures of treatment expectation, depression symptoms and treatment related side-effects will be assessed after baseline instructions and at 7 timepoints from 40 min to 1 week after treatment. Further information on methodological details are provided below.

#### Hypotheses

- The (main) effect of positive expectation (high/low) on emotional (face) processing will be associated with increased PFC and amygdala activity.
- We expect an additive main effect of expectation and medication at the behavioural and neural level, such that effects are most pronounced in the high expectation + verum group and lowest in the low expectation + placebo group (high + verum > low + verum ≥ high + placebo > low + placebo).
- Baseline activation in regions involved in emotion processing (in particular VMPFC, amygdala) will be predictive of 4-h and 1-week treatment effects.
- Baseline VMPFC and rACC functional (rsfMRI) and structural (DTI) connectivity with amygdala predicts placebo treatment response after 4 h [24].
- The impact of positive treatment expectation (high/low) on symptoms of depression will differ between subjective and objective outcome measures of depression (MADRS vs BDI).

#### Study 2: Reward processing

The second fMRI study will investigate how treatment expectation modulates activity in brain regions involved in reward processing in patients with MDD. Following the same expectation induction and treatment procedure as summarized above and detailed in “Experimental design,” the patients will perform a monetary incentive delay task (MID; [31]) during fMRI (see “fMRI paradigms” below and Fig. 1). Here the patients will be cued to anticipate and respond to a rapidly presented target in order to gain or avoid losing varying amounts of money. The same measures of treatment expectation, depressive symptoms and treatment related side-effects as described in Study 1 will be employed.

#### Hypotheses

- Positive treatment expectation (high/low) in MDD will result in enhanced VMPFC activation during reward processing.
- Baseline activation in regions of the mesolimbic system during reward processing (in particular hippocampus, NAc) will be predictive of 4-h and 1-week treatment effects.
- High vs low treatment expectations in MDD will differentially affect VMPFC activation during reward processing.
- More specifically, we expect that the interaction of treatment expectation and treatment indicates most pronounced effects in the high expectation + verum group and lowest in the low expectation + placebo group (high + verum > low + verum ≥ high + placebo > low + placebo).
- Baseline VMPFC and rACC functional (rsfMRI) and structural (DTI) connectivity with hippocampus predicts placebo treatment response after 4 h [24].

#### Experimental design

##### Factor 1 Medication (esketamine vs placebo)

The study drug will be provided as a solution for infusion/injection. Each device delivers either esketamine hydrochloride (0.25 mg/kg of esketamine) or placebo (sodium chloride 0.9%). Placebo and verum treatment will be provided via identical devices which will be prepared and delivered by a dedicated clinical trial pharmacy not involved in any of the other procedures in the trial. None of the patients, research staff or clinical staff will be informed about the patient assignment throughout the trial. Blinding will also be maintained throughout the statistical analysis. This has proven the most efficient dose with tolerable side effects in a previous trial [24]. Details on the procedure used in this trial can be found under the following link: https://www.sciencedirect.com/science/article/abs/pii/S0165032719319780?via%3Dihub.

Patients will be monitored for 4 h in the Department of Psychiatry, Philipps-Universität Marburg following administration, including continuous vital signs monitoring (i.e. heart rate, blood pressure, respiration and pulse oximetry).

##### Factor 2 Expectation (high/low)

While all patients will be correctly and non-deceptively informed that they are going to be randomized to placebo or verum, treatment expectation will be varied by providing incorrect probabilities for this randomization. In the high expectation group, participants will be (deceptively) informed that the study medication most likely (90%) contains the active drug, while the low expectation group will be (deceptively) informed that it is very unlikely (10%) that the study medication contains the active drug, but placebo [34]. One hour after expectation induction and receiving the study medication, patients will undergo MRI measurements: T1, DTI, fMRI resting state and two experimental “fMRI paradigms” addressing emotional and reward processing: (1) implicit processing (gender judgement) of positive and negative emotional faces (emotion processing) and (2) reward processing [31]. The paradigms are chosen because they (i) investigate emotional-cognitive-behavioural processes relevant for expectation formation and they reliably activate brain networks implicated in (ii) MDD, (iii) its treatment effects and (iv) placebo response in previous studies. Psychometric assessments at baseline will include BDI, MADRS, YMRS, expectation scale, dissociative experience scale and GASE. After pharmacological challenge assessments will include BDI, MADRS, YMRS, dissociative experience scale, GASE at 40 min, 4 h, 24 h, 3 days (+ expectation scale) and 7 days (+ expectation scale) after treatment (cf. “Data collection and management” section; adapted versions for symptom assessment at present will be used for assessments from 40 min to 3 days post-dose.) Treatment outcome in response to expectation and drug treatment will be assessed at the behavioural, subjective and neural level. After the end of the study (day 7 after final study assessments), the participants will be informed about the deception.

#### fMRI paradigms

##### Study 1: Emotion processing task

This modified version of the task used by Surguladze et al. [9] involves the presentation of grayscale faces with happy or fearful expressions in 16.8-s blocks of 10 pictures each, with each picture presented for 1.5 s, followed by 200 ms blank screen. There will be sixteen 16.8-s blocks with facial expressions (10 fear, 10 happy), and each block will be followed by a 16.8-s baseline block during which a fixation cross will be presented (ACBC block design). The face stimuli (60 individuals, each showing the two different emotions) were selected from the Karolinska Directed Emotional Faces (KDEF) dataset (http://www.emotionlab.se/resources/kdef). On presentation of each facial expression, patients will be required to indicate whether the face is a male/female by pressing the left (female)/right (male) button on a button box, simply to ensure they are attending to the stimuli. During baseline, the patients will be instructed to press the button for each cross presented for 1.5 s, followed by 200 ms blank screen.

##### Study 2: Reward processing

Patients will perform a “Monetary Incentive Delay” (MID) Task [31]. Each of two MID task runs consist of 90 6-s trials, yielding a total of 180 trials. During each trial, patients will see one of nine cue shapes (cue; 250 ms), fixate on a crosshair as they wait a variable interval (anticipation; 2000–2500 ms), and then attempt to respond with a button press during the presentation of a white target of variable duration (target; 80–360 ms). Feedback (outcome; 1650 ms) will follow the disappearance of the target, which will notify subjects how much money they had gained or lost that trial as well as their cumulative total up to that point. On incentive trials, subjects can either gain or avoid losing money by pressing the button during target presentation. Task difficulty will be based on reaction times collected during a practice session prior to scanning and set such that participants will succeed on approximately 66% of their target responses. Cues signal potential gains (*n* = 72, denoted by circles), potential losses (*n* = 72; denoted by squares) or no response requirement (*n* = 36; denoted by triangles). Gain cues signal the possibility of winning 0.00 € (*n* = 18; no lines), 0.20 € (*n* = 18; one horizontal line), 1.00 € (*n* = 18; two horizontal lines), or 5.00 € (*n* = 18; three horizontal lines). Similarly, loss cues signal the possibility of losing 0.00 € (*n* = 18; no lines), 0.20 € (*n* = 18; one horizontal line), 1.00 € (*n* = 18; two horizontal lines), or 5.00 € (*n* = 18; three horizontal lines). “No response” trials (*n* = 36; a triangle) indicate that the subject should not respond during that trial, and instead should wait until the cue signaling the next trial appears. Trial types will be pseudo-randomly ordered within each run and runs will be counterbalanced across subjects. Patients will be trained for at least 10 min and tested for explicit cue comprehension. After the trial, the patients will receive the money they have won during the task on top of their participant’s reimbursement.

#### Psychometric measures

All participants will undergo a standardized assessment of sociodemographic and psychological trait and state characteristics using paralleled instruments. These include acute and chronic stress levels, negative affect, anxiety and depression. Further, an individual’s treatment expectation and prior treatment experiences in general and with antidepressant treatments in particular will be assessed. A description of these measures is described in the “Data collection and management” section. At the study-specific level, assessment of depression symptoms (BDI, MADRS; adapted versions referring to symptoms at present will be used for assessments from 40 min to 3 days post-dose) will be taken repeatedly as part of the treatment efficacy assessment (on day 1 (predose and 40 min post dose), 4 h, 24 h, 3 days, 7 days, Fig. 1). Study-specific treatment safety assessments will include monitoring of adverse events (ECG, blood pressure, blood oxygen level) and treatment related side effects using the Clinician Administered Dissociative States Scale (CADSS; [35]) and Young Mania Rating Scale (YMRS; [36]) on day 1 (predose, 40 min, 2 h and 4 h post dose; adapted versions referring to symptoms at present will be used for assessments from 40 min to 3 days post-dose).

### Criteria for discontinuing or modifying allocated interventions {11b}

The intervention will be discontinued in response to harms, participant withdrawal of consent, or worsening condition.

### Strategies to improve adherence to interventions {11c}

No strategies to improve adherence to interventions have been put in place as the intervention consists of a single dose only.

### Relevant concomitant care permitted or prohibited during the trial {11d}

Best medical care according to guidelines will be given to all patients. The proposed study will not interfere with the patients’ treatments. Patients are treated according to S3 guidelines for treatment of depression. This includes both medication and state-of the-art psychological therapies.

### Provisions for post-trial care {30}

Following administration of the study medication, patients will be monitored for 4 h in the Department of Psychiatry, Philipps-Universität Marburg including continuous vital signs monitoring (i.e. heart rate, ECG, blood pressure, respiration and pulse oximetry). No provisions for post-trial care planned. For all patients in this trial, an insurance covering possible damage to the patients and an accident insurance is contracted at the Gerling Industrie Versicherung AG, Insurance number: 57 010312 03019/03262. This patient insurance covers any damage to health arising from participation in the study up to a maximum sum. In order not to violate the insurance cover, the patient must immediately notify the insurance company or the investigator in case of any damage to health arising from participation in the clinical study. A copy of the complete insurance terms and conditions will be made available to the patient. In addition the accident insurance covering accidents on the way to and from the study centre.

### Outcomes {12}

Primary outcome measures include:

- Brain activation in networks implicated in (i) reward and (ii) emotion processing, (iii) placebo and (iv) antidepressant treatment response in MDD and (v) the pathophysiology of MDD (in particular VMPFC, rACC, amygdala, hippocampus).

Secondary outcomes include:

- Behavioural reaction times and hit rates in the Monetary Incentive Delay task (see Study 2: Reward processing)
- Expectation-related changes in depressive symptoms (self-rating BDI, expert rating MADRS) in response to placebo and esketamine.

Primary outcomes will be assessed 1 h post treatment, secondary outcomes at 1h, 2h, 4h, 24 h, 3days and 7days post treatment.

### Participant timeline {13}

The SPIRIT participant timeline for the study can be seen in Table 1.

### Sample size {14}

Performing power calculations for voxelwise imaging data is problematic because of the large number of measures examined (typically > 100,000 voxels). In addition, even with appropriate correction for multiple comparisons, effect sizes from voxel-wise imaging data are inflated leading to erroneous estimates of required sample sizes. Thus investigators in the field often use previous studies as a guide to the number of subjects needed to recruit. To date, only very few previous studies [30, 32, 33] with sample sizes ranging between *N* = 10 and *N* = 37 have used fMRI to investigate the effects of i.v. ketamine on the functioning of neural networks related to reward processing in 10 patients with treatment-resistant MDD (cf. our primary outcomes; [30]). This study found that the improvement in mood was accompanied by an increased recruitment of the orbitofrontal cortex, ventral striatum, medial substantial nigra and ventral tegmental area, structures that are part of the reward circuitry. However, these studies either did only include patients with treatment-resistant MDD [30, 33], no placebo condition [30] or only included patients in remission [32]. A recent cross-sectional fMRI study on the effects of ketamine vs placebo on the processing of happy vs angry facial expressions in patients with MDD [27] reported robust effects in brain regions relevant for emotion processing (particularly the amygdala) with 33 patients which is comparable to the numbers we are planning to recruit in our study (4 groups, each comprising 36 patients each + 20% dropout = 4 × 44 patients = 176 patients). This sample size calculation is substantiated by a power analysis of behavioural effects (cf. our secondary outcomes): To be on the conservative side, we have powered the present study to detect small-to-medium effect sizes (*d* = 0.40) of the expectation manipulation including interactions with behavioural reaction times and hit rates in the Monetary Incentive Delay task (see Study 2: Reward processing), assuming an alpha of 5% and a power of 90% which results in a required sample size of *N* = 36 per group (4 groups, each comprising 36 patients each + 20% dropout = 4 × 44 patients = 176 patients).

### Recruitment {15}

A total of 176 patients with MDD according to DSM-5 criteria will be recruited from in- and outpatient settings from the Department of Psychiatry and Department of Psychology, University of Marburg, the Department of Clinical Psychology, University of Marburg and surrounding psychiatric hospitals. This monocentric study will be conducted as part of the Collaborative Research Center (CRC 289) “Treatment Expectation: The impact of expectation on health outcome” at the Department of Psychiatry and Psychotherapy, Philipps-Universität Marburg. The department has adequate staff and experience in treating patients with MDD and in conducting clinical trials as well as experienced physicians and supportive staff with adequate time, the targeted patient population and technical expertise to complete the protocol. It has a track record in recruiting patients with MDD, first episode psychosis, schizophrenia, anxiety disorders and bipolar disorder to large-scale national and international single- and multi-centre studies involving neuroimaging, deep phenotyping, clinical and cognitive assessments (FOR2107, Panic-Net I, Panic-Net II, PROTECT-AD, BipoLIFE, PSYSCAN, among others).

## Assignment of interventions: allocation

### Sequence generation {16a}

Patients will be randomized into one of the four experimental conditions (*n* = 4 × 44) using a balanced randomization procedure using block randomization. The block size will be randomly varied to reduce the likelihood of foreknowledge of treatment assignment among those recruiting participants.

Randomization and allocation sequence will be performed centrally by the central office of the Coordinating Center for Clinical Trials in Marburg (Koordinierungszentrum für klinische Studien (KKS/CCCT). Variations in block sizes will be performed by the KKS unbeknownst to those who enroll participants or assign interventions. The randomization of an eligible patient can take place if all inclusion criteria and none of the exclusion criteria are fulfilled. Therefore the investigator completes the study specific randomization form, which is part of the Investigator Site File (ISF), and sends it to the KKS Marburg via fax. The chance for allocation to the 4 groups is 1:1:1:1. The KKS reports the randomization result in form of a “medication number plus expectation induction” back to the centre. In the following, the pharmacist at centre has to note the centre-specific patient pseudonym on the medication and the emergency envelope which are already labeled with the corresponding package number.

### Concealment mechanism {16b}

The allocation sequence will be provided centrally by the Coordinating Center for Clinical Trials (KKS) in Marburg with none of the investigators or other study personnel at site involved.

### Implementation {16c}

Eligible participants will be enrolled in the study by the study team. The allocation sequence will then be generated on request by the Coordinating Center for Clinical Trials in Marburg who will also assign participants to the interventions.

## Assignment of interventions: blinding

### Who will be blinded {17a}

None of the patients, research staff or clinical staff will be informed about the patient assignment throughout the trial. Blinding will also be maintained throughout the statistical analysis.

### Procedure for unblinding if needed {17b}

Unblinding may occur for emergency purposes only. Investigators should note that the occurrence of a serious adverse event should not routinely precipitate the immediate unblinding of the label. If the treating physician considers it necessary to unblind the study medication in case of an adverse event, the emergency envelope can be opened—if possible, after prior contact with the KKS Marburg. The KKS must be contacted within 24 h after unblinding and the Unblinding Form (in the ISF) has to be faxed to the KKS Marburg. The date and the event making it necessary to unblind the treatment randomization have to be documented in the patient files and in the CRF. After the end of the trial, untouched envelopes must be returned to the KKS Marburg.

## Data collection and management

### Plans for assessment and collection of outcomes {18a}

The sequence of data collection can be found in Table 1 (cf. 13) and Fig. 1 (cf. 11a).

The following standardized and specific study information will be collected from all patients:

- *Numerical Rating Scale (NRS)* for a generic assessment of treatment expectations, pretreatment experiences (dose, quality [positive/negative], discontinuation effects), and treatment outcome (Rief et al., unpublished reports). Specific anticipated effects of a therapeutic intervention will be assessed using a numerical rating scale (NRS, 0–10) whenever appropriate. This will be complemented with generic assessments on pretreatment experiences and general clinical outcome measures. Repeated measures of expectations (i.e. at least two respective measures pre- and post-manipulation or intervention) will allow us to analyse stability, variance and changes of treatment expectations over time.
- *Treatment expectation*: Treatment Expectation Questionnaire TEX (15 items; [37]). We will also use this self-rating scale assessing treatment expectation with greater complexity than single NRS items.
- *Anxiety, negative affect, stress: State-Trait-Anxiety-Depression Inventory* (STADI; 40 items [38]). The most widely used instrument to assess state and trait anxiety is the STAI [39]; however, this instrument does not clearly discriminate anxiety and other dimensions of negative affect. An alternative was recently suggested that offers separate scales for anxiety and depression while still enabling the differentiation of state and trait aspects: the State-Trait-Anxiety-Depression-Scale STADI [38]. Anxiety is deconstructed into nervousness and worrying, while depression consists of the two factors dysthymia (negative affectivity) and anhedonia.
- *Behavioral Approach System Sensitivity* (BIS BAS, 24 Items; [40]). Personality traits linked to individual differences in reward processing, such as behavioural approach system (BAS) sensitivity or the agency facet of extraversion, have been consistently linked to individual neurophysiological differences, e.g. in the dopamine system. Considering the close link between dopamine release, reward-driven learning, selective attention, and expectation effects on treatment outcomes, the BAS scale of the BIS/BAS questionnaire will be used to gain new insights into the relationship between interindividual differences in dopaminergic “pathways” and expectation effects on treatment outcome.
- *Perceived Stress Scale* (PSS-10, 10 Items; [41]). An individual’s acute stress level will be assessed using the Perceived Stress Scale in its 10-item version, which mainly focuses on psychophysiological stress aspects.
- *Somatosensory Amplification Scale* (SSAS, 10 Items; [42]). Members of the CRC 289 have provided initial evidence for the predictive role of somatosensory amplification in the development of side effects/nocebo effects [43]. An individual’s tendency to reveal somatosensory amplification will be assessed using the SSAS.
- *Side effects*: Generic Assessment of Side Effects (and somatic symptoms in general) GASE (34 Items; [44]). Side effects of clinical and experimental therapeutic interventions will be assessed by applying the Generic Assessment of Side Effects Scale GASE. This scale assesses the 33 most frequent side effects in clinical trials and is based on the statistics of the Food and Drug Administration, USA and other surveys. Population based reference data of a sample of more than 2500 persons are available
- *Disability*: Adaptation of the pain disability index (PDI) (7 items; [45]). This instrument used to be widely used in pain research, but was adapted to be also suitable for disability associated with symptoms in general. The modified version has been evaluated in a sample with 2500 participants, which also provides a robust basis for normative data.
- *Personality*: Big Five Short Screener (BFI-10, 10 Items; [46]). Results indicate that the BFI-10 scales retain adequate levels of reliability and validity. Reducing the items of the BFI-44 to less than a fourth yielded effect sizes that were still sufficient for research settings with large samples and limited time constraints.
- *Montgomery Asberg Depression Scale* (MADRS; [47]): This is an expert interview to assess depression severity (patient groups). In contrast to the frequently used Hamilton Depression Scale, it is closer linked to the clinical features and classification criteria of DSM depression diagnosis.
- *Assessment of mental disorders* with SCID-Interview for DSM-5 [48].
- *Beck Depression Inventory BDI-II* [49]. This is the most frequently used depression self-rating scale worldwide.

### Plans to promote participant retention and complete follow-up {18b}

Participants will be given 170€ at completion of the post-assessments plus the sum they won during the MID-task in Study 2 (max. 73,65€) on day 28, regardless of whether they are allocated to the intervention or control arm.

### Data management {19}

The trial will use an electronic case report form (e-CRF/EDC-System) for data collection and documentation, which is hosted by KKS Marburg. The data are entered directly via web browser to the e-CRF and are transferred via encryption (HTTPS (TSL/SSL)) to the central database. Access to the e-CRF is only allowed for persons who are documented as trial personnel. Each person who is allowed to make entries in the e-CRF receives a personal username and the URL for database login upon request (User-ID request). The initial password, which has to be changed at first login, is transmitted automatically by email to the user upon request (Forgot Password?) to the personal email address, which is recorded in the system. Before a user gets access to the productive environment, the user account is only activated for training. After the user has activated its account, the user management at KKS enables the user for the appropriate site. The access level in the e-CRF depends on the group membership (investigator, study nurse, monitor, etc.). Thus, it is ensured that only authorized persons have access to the EDC system in order to document or monitor patient data. Users with monitoring function are not able to enter or change patient’s data. They have the possibility to view the data write protected (review function) and they can use additional review functionality in case of any implausibility or questions/queries. The completed e-CRF must be electronically signed (authorization) at the end of each visit by an investigator for each patient. In addition, a final verification of a case form for each patient has to be performed by the principal investigator or the substitute. This final verification confirms that the patient’s case report form is completely and accurately documented and reviewed by the investigator. In order to ensure the anonymity of the patient data, the patient data in the e-CRF are recorded with a patient number consisting of a centre number and a consecutive number. An allocation list (e.g. Rando-Log) containing the patient’s number and the identifying data of the patient is only kept in the centre. Users of the EDC system receive training material (EDC Manual), which is provided by the KKS. The EDC-Manual is part of the ISF and contains detailed instructions for using the EDC system. If necessary, KKS Marburg will provide additional training material and required documentation for the users. For training purpose of data entry and data review, a training site is included in the database.

In a multistage procedure, the given data will be checked electronically for their plausibility and consistency. Even during data collection, implausible data will be flagged automatically by implemented validation checks. Detected inconsistencies and missing or implausible data will be clarified with queries (electronically or paper-based) and necessary changes will be carried out. The EDC system has an implemented audit trail. This assures that any documentation and/or changes to database items are traceable anytime. At the end of trial, the database will be closed after data cleaning process. This process will be documented according to SOPs of KKS Marburg. The pseudonymized patient data recorded in the e-CRF are stored by the KKS Marburg in accordance with legal requirements.

All FMRI data will be collected and stored at the Psychiatry Marburg site for project-specific analysis. Neuroimaging and psychometric data will only be stored using participants’ pseudonyms. Access to all data will only be provided for documented trial personnel, individuals not involved in the study will not be able to access the data.

#### Source data and subject files

The investigator has to keep a written or electronic subject/patient file for every subject participating in the clinical study. In this file, the available demographic and medical information of a subject has to be documented, in particular the following: name, date of birth, sex, height, weight, subject history, concomitant diseases and concomitant drug (including changes during the study), statement of entry into the study, study identification, subject number, the date and process of informed consent, all study visit dates, predefined performed examinations and clinical findings, observed AEs (if applicable), and reason for withdrawal from the study if applicable. It should be possible to verify the inclusion and exclusion criteria for the study from the available data in this file. It must be possible to identify each subject by using this patient file. Additionally, any other documents with source data, especially original printouts of data that were generated by technical equipment, have to be filed. All these documents have to bear at least subject identification and the printing date printed by the recording device to indicate to which subject and to which study procedure the document belongs. The medical evaluation of such records should be documented as necessary and signed/dated by the investigator. Computerized subject files will be printed whenever source data verification is performed by the monitor. Printouts must be signed and dated by the investigator, countersigned by the monitor and kept in a safe place.

For the current study, documents considered to be source data include (but are not limited to):

- Subject’s record (subject’s clinic and/or office chart, hospital chart).
- Patient Informed Consent Form
- Laboratory results
- Pharmacy records
- Treatment notes
- Scores
- Any other records maintained to conduct and evaluate the clinical study

### Confidentiality {27}

In this trial the “REGULATION (EU) 2016/679 OF THE EUROPEAN PARLIAMENT AND OF THE COUNCIL of 27 April 2016 on the protection of natural persons with regard to the processing of personal data and on the free movement of such data, and repealing Directive 95/46/EC (General Data Protection Regulation)” will be noted by all parties involved.

### Plans for collection, laboratory evaluation and storage of biological specimens for genetic or molecular analysis in this trial/future use {33}

See above 26b there will be no biological specimens collected.

## Statistical methods

### Statistical methods for primary and secondary outcomes {20a}

fMRI data will be analysed using SPM12 software (Wellcome Department of Cognitive Neurology, London, UK) and appropriate toolboxes. Standard pre-processing steps include realignment, motion correction, high- and low-pass filtering, correction for temporal autocorrelations, normalization and smoothing.

We will analyse task-related fMRI data using the general linear model (GLM) approach. We will investigate the main effects of medication (esketamine vs placebo) and positive expectation (high vs low) using flexible factorial models focusing on predefined regions-of-interest (ROI) including the amygdala, hippocampus, insula, thalamus, ACC/PCC, dorso- and ventrolateral PFC, precuneus, caudate and Nacc (ROIs cf above Functional neuroimaging of emotion and reward processing in MDD). In Study 1 (Emotion processing), we are planning to compare BOLD responses to emotional faces and baseline fixation crosses on a single subject level, using appropriate contrasts that will then be included to a second GLM (group level). In Study 2 (Reward processing), four separate orthogonal regressors will be designed to contrast responses to gain and loss during anticipation and outcome versus neutral conditions (i.e. no-gain and no-loss anticipation and outcome): gain versus no-gain anticipation (GVNant), loss versus no-loss anticipation (LVNant), gain versus no-gain outcome (GVNout), and no-loss versus loss outcome (NVLout) (as used previously; [31]). Generally, based on our hypotheses, a small volume regions of interest (ROI) approach with family wise error correction (FWE) will be applied using predefined anatomical ROIs based on the Harvard Oxford atlas and meta-analyses conducted on the neurosynth.org platform. Relevant variables (e.g. age, gender, treatment expectation scores, type of antidepressant medication other than esketamine [i.e. SSRI, SNRI, other AD, combination pharmacotherapy], severity of depression symptoms) will be included as covariates to control their influence on changes in neural activity in the different experimental phases or to test for their relationship with relevant neural processes (reward processing, emotion processing). Post hoc analyses will explore the effect of type of AD medication. Exploratory analyses will comprise whole brain analyses. In addition to task-related data, functional and structural connectivity will be analysed in order to address the relationship between inter-individual differences in intrinsic functional connectivity and structural connectivity/integrity and expectation-modulation and emotion and reward-processing networks. These analyses will focus particularly on hippocampal-medial prefrontal-amygdala circuits. Morphometric analyses will focus on hippocampus, insula, sgACC and amygdala ROIs and correlate these with treatment outcome. Finally, we will use voxel-based morphometry (VBM) to test for an association between structural predispositions in the abovementioned ROIs and behavioural measures of expectation effects on depression symptoms.

### Interim analyses {21b}

There are no interim analyses planned.

### Methods for additional analyses (e.g. subgroup analyses) {20b}

There are no subgroup analyses planned.

### Methods in analysis to handle protocol non-adherence and any statistical methods to handle missing data {20c}

Missing data will not be imputed. All available data will be used in the models.

### Plans to give access to the full protocol, participant-level data and statistical code {31c}

Individual participant data will be shared with the study team of the CRC/TRR 289 after deidentification and will be available in this form for other researchers upon reasonable request. Only anonymized data in agglomerated form is used for publications. No personal data will be shared.

## Oversight and monitoring

### Composition of the coordinating centre and trial steering committee {5d}

The coordinating centre is placed at the Department of Psychiatry and Psychotherapy, University of Marburg, Germany. The day-to-day management of the trial will be handled by the Trial Coordinator and the Principal Investigators in collaboration who meet on a weekly basis with the other members of the study team who are responsible for trial set-up, administration and recruitment. These other members include study physicians, study psychologists and research assistants. The Trial Steering Committee consists of the Principal Investigators. The Steering Committee has additional meetings on a monthly basis (and as needed) to oversee the trial by reviewing and approving the study progress.

### Composition of the data monitoring committee, its role and reporting structure {21a}

The monitoring of the study will be carried out by monitors of the trained staff of KKS Marburg. Patient recruitment can begin after the initiation visit. During the course of the study, the site will be visited for monitoring before, during and after the study. During each of these visits, source data verification will be performed on the basis of a prespecified sampling plan, generated by the KKS Marburg.

The following patient data have to be verified 100% by all means:

- Patient identification
- Patient informed consent
- Major in- and exclusion criteria
- Adherence to the randomized therapy
- Serious adverse events
- End of trial

In general, any discrepancies in the CRF will be discussed and clarified with the study team during the monitoring visit and corrections/additions will be made according to GCP requirements. Furthermore, problems will be discussed at these visits. Source data verification will be performed by direct access to the original patient records. The institution responsible for monitoring guarantees that patient confidentiality will be respected at all times. Participation in this study will be taken as agreement to permit direct source data verification.

### Adverse event reporting and harms {22}

All adverse events (AE) occurring after signing the informed consent form must be reported up to 28 days after the last dose of study medication was administered. After that time, only serious adverse reactions (events, possibly related to study medication) have to be reported. For the purpose of SAE reporting, the study specific reporting form has to be used. It is the responsibility of the investigator to fax all SAEs within 24 h of becoming known to: Philipps-Universität Marburg,KKS-Marburg.

### Frequency and plans for auditing trial conduct {23}

In compliance with European regulations/ICH-GCP Guidelines, it is required that the investigator and institution permit authorized representatives of the sponsor and the regulatory agency(ies) direct access to review any study-related documents and subject’s original medical records for verification of study-related procedures and data during and/or after the study. The extent is permitted by the applicable laws and regulations. By signing a written informed consent form, the subject or the subject’s legally acceptable representative is authorizing such access. Direct access includes examining, analysing, verifying and reproducing any records and reports that are important to the evaluation of the study. The investigator is responsible for giving any requested support for any monitoring, inspection or audit visit. The Principal Investigators have to be available during these visits.

### Plans for communicating important protocol amendments to relevant parties (e.g. trial participants, ethical committees) {25}

Any substantial amendments to the protocol will be submitted to the ethical committee. Updates are made to the trial registry when required.

### Dissemination plans {31a}

The results will be published in an international peer-reviewed journal with open access and disseminated as conference presentations. If the results from the trial have public interest, they will also be presented to mainstream media.

## Discussion

Placebo responses in antidepressant trials have become a critical issue for the development of novel therapeutics and the treatment of patients with MDD in clinical settings. Increasing placebo response complicates efforts to detect signals of efficacy for new agents in the drug development setting, while at the same time clinicians know that many patients will not experience sustained remission of their depression with currently available treatments. Much of this discourse has been complicated by a lack of understanding of what contributes to placebo responses in MDD. The systematic investigation of the role of treatment expectation and its underlying neural mechanisms in MDD will provide the knowledge base to develop novel strategies to enhance the efficacy and tolerability of and adherence to antidepressant treatments. This study aims to provide such first insights which will lay the foundation for future studies in which we aim to identify factors relevant to generating treatment expectation in patients with MDD, including patient variables (e.g. chronicity of the illness, previous treatment experience, peer-influence, genetic variables) as well as prescriber-variables (e.g. interpersonal style, age) and information given to the patient (e.g. expected effects, side effects, biological mechanisms) in order to disentangle the contribution of placebo response to pharmacological response. The balanced placebo design using the fast-acting agent esketamine will open a new chapter in the pharmacology of major depressive disorder as it allows to test for additive or potentially synergistic effects of expectation and pharmacological agent. Future studies may also shed light on substance-specific differences in its additive or interactive potential with expectation effects. Taken together, this new knowledge will inform research aimed at reducing expectation effects in clinical trials as well as research aimed at increasing antidepressant efficacy in clinical practice. This may include the development of strategies to limit placebo response in the clinical trials setting and strategies to increase placebo response in clinical practice.

## Trial status

Protocol version 3.0 (May 2023). Recruitment commenced June 2021. Anticipated completion of recruitment is August 2023.

## Data Availability

Individual participant data will be shared with the study team of the CRC/TRR 289 after deidentification and will be available in this form for other researchers upon reasonable request. Only anonymized data in agglomerated form is used for publications. No personal data will be shared.

## Abbreviations

ACC: Anterior cingulate cortex
AD: Antidepressant
BDI-II: Beck Depression Inventory II
BOLD: Blood oxygen level-dependent
BRS: Brain reward system
CRF: Case report forms
DLPFC: Dorsolateral prefrontal cortex
DTI: Diffusion Tensor Imaging
ECG: Electrocardiogram
fT4: Free prohormone thyroxine
fT3: Free Triiodothyronine
fMRI: Functional Magnetic Resonance Imaging
FWE: Family wise error
GASE: General Assessment of Side Effects
GGT: Gamma-glutamyltransferase
GOT: Glutamic-oxaloacetic transaminase
GVNant: Gain versus no-gain anticipation
GVNout: Gain versus no-gain outcome
IM: Intramuscular
IV: Intravenous
KDEF: Karolinska Directed Emotional Faces
KKS: Koordinierungszentrum für klinische Studien in Marburg (Coordinating Center for Clinical Trials)
MADRS: Montgomery Åsberg Depression Rating Scale
MDD: Major depressive disorder
MEG: Magnetoencephalography
MID: Monetary Incentive Delay
MRI: Magnetic Resonance Imaging
MRS: Magnetic resonance spectroscopy
Nacc: Nucleus accumbens
NMDAR: N-methyl-D-aspartate receptor
NVLout: No-loss versus loss outcome
PCC: Posterior cingulate cortex
PFC: Prefrontal cortex
phMRI: Pharmacological magnetic resonance imaging
rACC: Rostral anterior cingulate cortex
RCTs: Randomized controlled trials
ROI: Regions-of-interest
rsfMRI: Resting state fMRI
SAE: Serious adverse event
SCID-5: Structured Clinical Interview for DSM-5
sgACC: Subgenual anterior cingulate cortex
SmPC: Summary of Product Characteristics
SNRI: Serotonin–norepinephrine reuptake inhibitor
SSRI: Selective serotonin reuptake inhibitor
TSH: Thyroid-stimulating hormone
T1: Spin-lattice relaxation time
VBM: Voxel-based morphometry
VMPFC: Ventromedial prefrontal cortex
YMRS: Young Mania Rating Scale
